# Caring for late preterm infants: public health nurses’ experiences

**DOI:** 10.1186/s12912-018-0286-y

**Published:** 2018-04-18

**Authors:** Genevieve Currie, Aliyah Dosani, Shahirose S. Premji, Sandra M. Reilly, Abhay K. Lodha, Marilyn Young

**Affiliations:** 1 School of Nursing and Midwifery, Mount Royal University, 4825 Mount Royal Gate SW, Calgary, Alberta T3E 6K6 Canada; 2 O’Brien Institute of Public Health, University of Calgary, Calgary, Alberta Canada; 3 Faculty of Nursing, University of Calgary, 2500 University Drive NW, Calgary, Alberta T2N 1N4 Canada; 4 Department of Paediatrics, Section of Neonatology, Alberta Health Services, Foothills Medical Centre, 1403 29th Street NW, Calgary, Alberta T2N 2T9 Canada; 5 Alberta Children’s Hospital Research Institute, Calgary, Alberta Canada; 6 Prenatal & Postpartum Services, Public Health Calgary Zone, Alberta Health Services, 1430, 10101 Southport Road SW, Calgary, Alberta T2W 3N2 Canada

**Keywords:** Infant, Premature, Community health care, Nurses, Public health, Evidenced-based practice

## Abstract

**Background:**

Public health nurses (PHNs) care for and support late preterm infants (LPIs) and their families when they go home from the hospital. PHNs require evidence-informed guidelines to ensure appropriate and consistent care. The objective of this research study is to capture the lived experience of PHNs caring for LPIs in the community as a first step to improving the quality of care for LPIs and support for their parents.

**Methods:**

To meet our objectives we chose a descriptive phenomenology approach as a method of inquiry. We conducted semi-structured interviews with PHNs (*n* = 10) to understand PHN perceptions of caring for LPIs and challenges in meeting the needs of families within the community. Interpretative thematic analysis revealed PHN perceptions of caring for LPIs and challenges in meeting the needs of families within the community.

**Results:**

Four themes emerged from the data. First, PHNs expressed challenges with meeting the physiological needs of LPIs and gave voice to the resulting strain this causes for parents. Second, nurses conveyed that parents require more anticipatory guidance about the special demands associated with feeding LPIs. Third, PHNs relayed that parents sometimes receive inconsistent advice from different providers. Lastly, PHNs acknowledged that due to lack of resources, families sometimes did not receive the full scope of evidence informed care required by fragile, immature infants.

**Conclusion:**

The care of LPIs by PHNs would benefit from more research about the needs of these infants and their families. Efforts to improve quality of care should focus on: evidence-informed guidelines, consistent care pathways, coordination of follow up care and financial resources, to provide physical, emotional, informational support that families require once they leave the hospital. More research on meeting the challenges of caring for LPIs and their families would provide direction for the competencies PHNs require to improve the quality of care in the community.

## Background

Canada has a preterm birth rate (2014–2015) of 7.8% [1]. In 2014–2015, Alberta’s preterm birth rate averaged 8.7%, and Calgary’s rate averaged 9.2% [2]. Infants born between 34 0/7 weeks and 36 6/7 weeks’ gestation, referred to as late preterm infants (LPIs), comprise approximately 75% of this population [3]. The Canadian Paediatric Society supports the early discharge of stable LPIs, providing they receive appropriate follow-up within 48 h by a community-based health care provider [4]. While LPIs and term infants are similar in terms of size and mature appearance, there are significant differences. When compared to full term infants, LPIs have an increased risk for several morbidities. Specifically, LPIs experience a higher risk of feeding difficulties [5, 6], excessive weight loss [5], hypoglycemia [5, 7, 8], hyperbilirubinemia [5, 7, 8], hypothermia and temperature instability [7, 8], respiratory distress syndrome [8], and sepsis [8]. These medical issues can persist after discharge from hospital, and result in LPIs having a high rate of emergency room visits and hospital readmission within the first two weeks of life [9–12].

In Alberta, once LPIs are discharged from hospital, their home care becomes the immediate responsibility of public health nurses (PHNs). Birth and early postpartum hospital records are provided for continuity of care. In Calgary, PHNs contact all parents often within hours of arriving home to offer nursing support [13]. This support ranges from one home visit or clinic appointment to a series of consultations as required on a need-to- basis. The consultations not only focus on the LPIs but also the parents, who require teaching and guidance to become increasingly self-sufficient. Therefore, PHNs need to have an excellent understanding of how the needs of LPIs differ from those of the term infant. In so doing, PHNs typically rely on their empirical knowledge and the guidelines used with term infants, with some modification [14].

This research study aims to understand the lived experience of PHNs in caring for LPIs and supporting families in the immediate postpartum period. These research findings identify what challenges PHNs experience while providing care for LPIs and their families in the community setting, and what additional resources and competencies PHNs require to improve the quality of care for LPIs and their families.

## Methods

As part of a larger study using a mixed methods approach [15], this article reports on one aspect of the research. Descriptive phenomenology was used as the method of inquiry to better understand the lived experience of PHNs and parents in caring for LPIs in the community setting [16, 17]. Descriptive phenomenology explores the structure of experience and consciousness within everyday life [17]. This article presents the results of the PHN experience. Other results considering the parent experience appear elsewhere [18, 19]. The study participants, at three postpartum community service sites, completed a demographic questionnaire. A purposive sampling method [17] was then used to recruit PHNs according to years of experience, location, and role. Ten PHNs with a range of clinical experience from 3 to 30 years participated in the study. Some PHNs, considered experts, functioned in the role of charge nurse, lactation consultant, and/or clinical nurse educator. The PHNs determined where and when to conduct the in-depth, face-to-face semi-structured interviews, which ranged from 60 to 90 min in length.

The research questions were: 1) What is your experience in caring for LPIs as a PHN? And 2) What challenges have you experienced in providing care to LPIs and their families?

Researchers (AD, SR) modified or added questions to explore topics as they emerged during the interviews. The process continued until the researchers determined they had reached informational saturation [20]. Interviews were recorded, transcribed verbatim, and checked for accuracy before analyses. Two researchers (GC, AD) numerically labeled the interviews, to preserve anonymity, before they independently analyzed and categorized the themes. Using interpretive thematic analysis, the researchers (GC, AD, SP) assigned codes to various elements that emerged from the transcribed text, then identified narrative ideas before re-organizing these ideas around central themes [21]. In turn, any patterns found in the codes and central themes and relationships, identified across participants and narratives [22], served to answer the research questions.

## Results

Four themes emerged from the data. First, the complexity and intensity of care required by LPIs makes stressful demands on caregivers and providers. Second, LPIs have specific feeding challenges when compared with term infants for which parents lack anticipatory guidance. Third, inconsistencies in care practices between health care providers is difficult for parents and PHNs to navigate. Due to the lack of evidence-informed research, providers come to rely on their empirical knowledge, which sometimes leads to contradictory beliefs that can confuse families or lead to inconsistent care. Finally, as with many health promotion activities, the financial costs of a home visitation program raise questions about its sustainability within a publicly funded system, which can frustrate the best efforts of PHNs. Consequently, health resource management and allocation of care of LPIs also appeared as a major area of concern.

### Theme 1: The stress of providing nursing care for LPIs: “they think they’re bringing home a term baby…so we’re the bearers of bad news”

Meeting the physical demands of LPIs and subsequent emotional needs on the parents represent the most common challenge for PHNs. LPIs have multiple health challenges because of their physiological immaturity. They include: feeding difficulties, hyperbilirubinemia, temperature instability and poor temperature regulation, and risk of sepsis. As PHN #8 stated: *“…first of all they definitely tend to usually have problems with higher levels of jaundice so we have to monitor them more carefully for that.”* PHN #5 expressed *“They’re not warm enough and they’re not feeding well enough and the moms maybe are not feeding them often enough”.* PHN #3 described other health challenges:

… yes [we see them] with weight, with poor weight gain, with poor feeding, jaundice – all of that…And so they are being admitted day 5, day 6 for phototherapy and we have been faithfully following them.

PHNs described feelings of angst and frustration, exasperated by limited time and resources available to PHNs, trying to address these complex and interrelated needs of LPIs and their parents. For example, PHN #5 explained: *“There is a lot of pressure to do a tremendous amount of teaching in a short period of time. A lot of which goes over their heads ‘cause they are tired and they don’t hear everything you say.”* PHNs question whether hospital staff inform parents of the specific needs of their LPIs or parents simply forget some instructions, as some parents have no recollection that LPIs can experience numerous challenges. This can result in parents not having the information required to address these challenges, which puts PHNs in the difficult position of breaking unwelcome news, often when a problem arises, to the chagrin of the parents. A PHN explained:

Sometimes I feel like we’re the bearers of bad news because in hospital everything seems to be going so well. And then you go out to the first visit and the jaundice is going up and the baby’s lost weight and output is kind of iffy…Things aren’t where they’re supposed to be at (PHN #2).

Consequently, some parents leave the hospital with the impression that *“… they’re bringing home a ‘term’ baby”* (PHN #5). Thus parents have no forewarning of the homeostatic and feeding problems associated with LPIs. Accordingly, this difference in perceptions must be addressed by PHNs. PHN #5 expressed her perception of how the baby was progressing which was different than the parents:

The problem that we see a little more often is when the ones come home straight out of hospital that are 36 weeks and they’re different, cause they haven’t been in the NICU yet and they haven’t had that experience so they believe that they’re bringing home a ‘term’ baby and often times they’re not feeding, or transferring milks as well but the mom thinks the baby is feeding and the baby is really not feeding so they’re missing some of the cues of that baby...

PHNs also conveyed that LPIs frequently resemble term infants in size and appearance and this can lead to confusion as they often require more care. PHN #9 explained:

…[Parents] know their baby’s early, but… some of them still go home like the next day with their baby, right. Especially if they have a larger late preterm infant like a 6 to 8 pound late preterm infant, which is not unheard of. I think many of them do not perceive their infant as being any different from a term infant.

Moreover, PHNs have to reduce parental anxiety in such situations:

But sometimes they [parents] don’t know [what to do]. …And they feel really bad when they do find out, and they realize… my baby’s not feeding and the jaundice is now, you know, up in the red zone (PHN #2).

When things are not going well, mothers: *“have a lot of guilt and you’re always trying to reassure them… It’s very upsetting for them…”* (PHN #2). Hence, in addition to helping parents of LPI manage a variety of often interrelated challenges and identify and implement appropriate care strategies, PHNs must also be able to identify conflicting feelings among parents and address these also.

### Theme 2: Feeding challenges for LPIs: “suck, swallow, breathe is complex for these babies”

PHNs reported how infants tire at the breast, with a poor suck reflex, and inadequate suck swallow coordination. *“Suck, swallow, breathe is complex for these babies. They don’t coordinate it very well. And they [parents] need to understand that and that’s the problem that we have”* (PHN #5). PHN #3 observed that “*typically what we see [are LPIs] sucking a few times at the breast and then falling asleep”* and mothers have difficulties *“recognizing [these] cues”* (PHN #2). These difficulties become problematic because, *“the parents often think that ‘oh, they’re done. They’re full, and in fact, they’re not; they’re just kind of exhausted”* (PHN #2). To ensure adequate milk intake, PHNs described the significant amount of time they spend with parents: *“…we can provide a tremendous amount of teaching, education, a lot of feeding support one on one, [with]hours of time”* (PHN #5). PHN #9 articulated: *“I will sometimes spend upwards of an hour just working on breastfeeding in the community or in the clinic.”* According to PHN #2 *“Well certainly often there’s feeding issues so that, that certainly often takes extra time... getting these babies to the breast and feeding effectively at the breast.”* The inability of the parents to assess these satiety cues could of course, lead to inadequate feeding and becomes a significant nursing practice issue.

In addition, most parents aspire to breastfeed their LPIs exclusively but this presents more of a challenge with LPIs due to immaturity.

A lot of times these moms want to go to full breast feeding so the challenge is being able to stay with the family long enough to get this baby fully breast fed, you know, to where the mother actually has a good relationship with you and you want to keep that going so that she can meet these goals (PHN #5).

However, it can prove difficult because the infants quickly become physically exhausted and can only breastfeed for less than several minutes before they tire *“...many late preterm babies really tire at the breast quite easily so they’re using up almost as much calories working to get the milk and then the calories they are getting”* (PHN #9). In addition, parents are not always aware that feeding is ineffective and this becomes the priority for the PHN despite lack of understanding from parents:

Parents sometimes think everything is going well and the baby’s feeding well. And [then] you observe a feed, and you realize, you know, that it’s not really going well. I’m not hearing adequate milk transfer. [Consequently] the weights gain’s not great (PHN #2).

PHNs relayed specific challenges associated with assisting parents with a range of feeding strategies (breastfeeding, bottle feeding and mixed feedings) and they felt very responsible for promoting correct information. PHN #7 described her experience with bottle feeding:

So I find that it can be a little bit stressful in that they’re [parents] not picking up those cues and so I’ll teach them...you know the baby is grimacing or the baby’s hand goes up, the baby’s turning away it means they don’t want any more.... So that can be frustrating for me …Why hasn’t anybody told you this…Your baby’s at risk of, you know, aspiration...

PHN #4 recalled that parents appear unaware of the developmental challenges associated with being a LPI: *“When the baby’s too tired at the breast, you need to give her more of a break.”* With bottle-feeding, PHNs noted gaps in parents’ understanding of infant cues. It seems that parents sometimes used a *“bottle that’s too fast and [the infant was] choking and flooding on the bottle and shutting down and not wanting to eat”* (PHN #5). In these circumstances, parents and infants appeared to work at cross-purposes. PHNs recognized that because of these feeding challenges, weight gain appeared sporadic and not as linear and progressive as with term infants. PHN #6 noted the growth was slow:

...you have to be sort of patient with these, with these families, and that there’s little gains and little successes and that they, you know, as long as there’s a step forward up then, that, you know, that’s good but it’s just that they need to take a little bit more time which, you know, and the reassurance to families that they’ll look at it as a journey, and that, um the days are long....

Such observations generally prompted *“a lot of follow-up”* (PHN #2) from PHNs and typically for a long period of time to ensure consistent weight gain, and exclusive breastfeeding. In this regard, PHNs evidently provide significant critical analysis of feeding and outcomes for these infants when providing follow up care. *“...I think they have in their mind that they need to catch their baby up, right? It’s always a race to catch up and that’s a shame. Just let the baby grow at its speed and enjoy the baby...”* (PHN #7).

### Theme 3: Inconsistencies between health care providers: “…there needs to be more consistent information”

PHNs report a lack of consistency in the advice that providers give parents. PHN #7 indicated *“…that’s a big concern for parents. That the information given in the hospital is different from the information we’re giving. So definitely there needs to be more consistent information.”* PHNs conveyed experiencing frustration when helping parents make the transition from the hospital to the home. Parents seemed to apply everything that the NICU nurses told them. *“It was really hard to get through to him [the father] the importance [of feeding] ‘cause he figured that we couldn’t do anything else for him cause the NICU nurses taught him everything they needed to know”* (PHN #4). Parents of LPIs cared for in the NICU *“typically find it quite difficult to go from that rigid, every 3-hour feeding schedule, to knowing when and how to transition or move, um, onto demand feeding”* (PHN #8). Some PHNs expressed that these inconsistencies in messages result from the lack of anticipatory guidance parents received from other health care professionals while in the hospital. *“They [parents] feel very overwhelmed”* (PHN #2) especially if uninformed about the likelihood of multifaceted health challenges. *“They don’t always absorb everything that you’re saying cause it’s a lot for them”* (PHN #2). The parents of LPIs “*really do struggle… [The] mother is exhausted by the time morning rolls around…then she’s got the rest of the family to look after”* (PHN #5).

From PHNs’ perception, what parents perceive as inconsistent care is in fact PHNs modifying care strategies relative to the LPIs’ inconsistent growth and development patterns. For example, feeding behavior and growth patterns can vary as shared by PHN #7:

…oh the nurse [in the hospital] said the latch looked good. …Well does it feel good? No it doesn’t? Well, it’s not supposed to hurt.…Well if she told you it was relevant two days ago, … but now on day six it’s no longer the case [because] of what I’m seeing in front of me with the baby’s numbers and how you’re doing and how you’re presenting. So based on my assessment, we’re going [to need] to have a new plan.

These differences can prove difficult for parents *“it makes them upset, it makes them feel undermined”* (PHN #3) when their infants’ needs change over time, and the care strategies of hospital or community nurses differ. Additionally, the lack of evidence informed practice guidelines or protocols developed for LPIs in the community added to the variability in the care that is provided. PHN #10 described the dilemma succinctly,

“…there’s not much out there [referring to guidelines] …so what do you compare them to? Do you compare them to the extreme premies or do you compare them to the term babies?”

Since they are closer in age to term infants, *“I think they [PHNs] tend to compare [LPIs]more to term children”* (PHN #10). Consequently, some PHNs treat LPIs like term babies while others rely on their own experiential knowledge. This practice contributes to various PHNs having different ideas about the direction of care for LPIs. PHN #4 indicated that she *“…would have done [things] differently than what the other nurse had done. Like she had just said that we would call her, but I would have seen that baby again.”* PHNs also discussed the need for a consistent caregiver to follow the family and follow through on care suggestions: “…I think it [having the same PHN follow up] would help*”* (PHN #2). The reason for this is because *“sometimes we only see them that one time and then someone else will follow up so you don’t really get to…see [the] whole thing”* (PHN #2). This lack of continuity of care can be detrimental to the LPI and families since *“you make this promise and then the next day they [another PHN] phone and they don’t fulfill that promise with the mother. They change it [the care plan] and that is a downward spiral”* (PHN #1).

Whereas care practices between RNs practicing in the hospital and home do vary, other health care professionals also contributed to the lack of consistency. PHN #5 shared her perspective:

Unfortunately, sometimes, what happens is they go to the family doctor, and the family doctor tells them everything is okay – see you in two months – and then we don’t get a chance to see them again. Because…the baby may not be feeding well, and we may have concerns, but sometimes it’s hard to get those families to buy in [to what we can offer].

PHNs also struggle with parental perception of hierarchy with health care professionals. *“…A lot of times the doctor’s word is the end word”* (PHN #5). This causes difficulties between the PHN and the parent if they have received conflicting advice from the PHN.

### Theme 4: Financial constraints on health promotion: “She had nobody. No vehicle…we might be bringing them into clinic, two, three days in a row and that’s pretty punishing for them”

The PHNs expressed concern about the financial constraints on public health funding and the implications for providing postpartum care to an increasing number of families. PHN #1 explained her dilemma as follows: *“if we bring them to clinic [it is more efficient because] more people [can be] seen in the day…”.* However, PHNs understand the limitations of a clinic visit, since the clinic setting does not offer the best setting in which to observe feeding practices. For this reason, they prefer to assess and counsel mothers in their homes. PHN #3 stated:

as a lactation consultant, I feel strongly that if I can see a mother in her own chair, with her own pillow, to look at her own comfort, in her own home, it is a very different experience from bringing them in to the clinic. And that helps enormously.

PHNs described a preference for delivering care in the home since it provides the opportunity to understand the family’s physical environment and social support network first hand: *“...when we are in the home we can see exactly what’s in the environment. We probably get a better idea of the social situation probably in their home...”* (PHN #8). Given their fragility, LPIs remain susceptible to environmental influences, and PHNs prefer a home visit to conduct a proper assessment and intervene more effectively. PHN #5 explained, *“The advantage of home visit is you’re not bringing out a new baby …putting her in a car or bringing her for a half-hour drive to see you… They’re sore, they’re tired, they don’t feel great, and they want to be at home.”* Furthermore, home visits are preferable *“…if it’s especially warm or really cool or really windy or really rainy day, [home visits] help prevent [any associated risks of] these changes in temperature for the babies”* (PHN #9). Finally, home visits are preferable since *“…they’re[parents] comfortable in their setting. They’re more relaxed in their [home] setting and they can speak freely”* (PHN #5).

PHN #1 stated her rationale for home visits quite convincingly:

…When you go to their home, it’s easier to see if their living conditions are very poor. If you get to the front door and the screen is all ripped out and there are cigarette butts everywhere outside the front door and beer bottles, it’s a different situation than as if they have been asked to come to the clinic and so you need that 2 h to get into even[assess] the finances and start resources for the mother that have not been really worked on enough in the hospital.

Some PHNs voiced concern for families with serious transportation problems. For them, access became an obstacle. In the case of one family: *“She [the mother] didn’t have any other family in Calgary... She had nobody. No vehicle, no access to a vehicle”* (PHN #2). The demands could become even more difficult if *“…we are seeing them the next day and we might be bringing them into clinic, two, three days in a row and that’s pretty punishing for them…”* (PHN #3). For many vulnerable families, home visitation would more likely ensure that parents and LPIs receive the quality of care required.

## Discussion

This study represents the first in-depth exploration of PHNs’ lived experience of caring for LPIs in the community. The research findings describe how the lack of evidence informed guidelines, consistent care pathways, coordination of care, and general resources affected nursing care.

PHNs shared their experiences surrounding the care of LPIs in the community and how the vulnerability and fragility of LPIs requires special attention. Multifaceted health challenges include: temperature instability and jaundice, as well as feeding difficulties. These physical challenges relate to the developmental and neurological immaturity of the infant [8, 10]. This becomes problematic for health care providers when there are not specific care guidelines tailored to LPIs experiencing such challenges. Narratives from PHNs told how in the absence of such guidelines, providers and caregivers often treat LPIs as term infants. This thinking reinforces misunderstandings about differences in growth parameters, and how feeding problems related to choking and aspiration, as well as jaundice and temperature instability are sometimes under assessed for LPIs. Our findings suggest PHNs require evidence informed care and feeding guidelines when caring for LPIs. Such an approach has worked successfully in the hospital setting [22].

Home care guidelines would particularly benefit those families of LPIs discharged early [14]. Reyna, Pickler, and Thompson [23] in their study of early preterm infants transitioning home from the NICU environment, suggest focusing on common feeding issues, such as suck swallow breath coordination as well as hunger and satiety cues. Since LPIs face similar challenges, guidelines would apply to them as well. Other researchers have proposed strategies and resources for health care providers working with premature infants. The researchers recommend that providers address the psychological strain parents can experience when caring for LPIs, including anxiety and apprehension relating to feeding difficulties, and the lack of personal confidence to provide the appropriate level of care at home [24–29].

The lack of evidence informed guidelines for LPIs may lead to inconsistent care by health care professionals. These inconsistencies can distress and confuse parents. Boykova [30], Russell and colleagues [31], and Boykova and Kenner [32] found similar results highlighting that parents require nurses with specialized knowledge of the preterm infant and discrepancies in care amongst nurses may not only affect care outcomes, but ultimately results in a lack of confidence and trust in the health care provider.

Our findings suggest that acute care providers in the hospital need to prepare parents for the unique challenges of caring for a LPI. The need for meticulous attention to discharge planning is congruent with a synthesis of literature. Adama, Ayes and Sundin [29]**,** Boykova [30], Griffin and Pickler [26], and Jefferies [33] collectively recommend that preterm infants require early intervention in discharge planning well before the date of discharge. It is important for discharge planning to include assessing parental knowledge barriers surrounding care of the preterm infant. Griffin and Pickler [26] go further to recommend that prior to discharge, nurses should provide a structured and individualized discharge plan, including assessment of suitability for discharge, based on the infant’s needs, parental competencies, resources, and risk factors. Furthermore, Boykova [30] suggests conducting an assessment of parents’ emotional state as well as the availability and quality of social supports available prior to discharge. Boykova [30] emphasizes that parental learning needs change over time. During the initial period following discharge, parents require information about care giving and medical conditions. Over time, these learning needs change and parents need information regarding growth and development. Hence, it is necessary for PHNs to consider how learning needs evolve and adapt their parental support measures accordingly.

Finally, financial constraints continue to restrict the allocation of resources for PHNs to care and coordinate interventions for LPIs and their families. Time constraints and limitations on the number of visits requires communication and continuity of care with all providers that care for LPIs. An interdisciplinary approach to care, from hospital to home, involving PHNs, physicians, and other health care professionals can be organized into a model of care specifically for LPIs. Follow up care using such care teams for seamless transition into the community is well supported in the literature to address vulnerable populations, including early and very early preterm infants transitioning from NICU [34–36] and medically fragile infants [37, 38].

Our study focused on postpartum community services in Calgary. We interviewed PHNs once during the study and did not return our analyses to them for member checking. We did, however, present our findings to PHNs who agreed with our findings. We did not triangulate the PHNs’ perspectives about parents of LPIs with parents’ experience of caring for LPIs.

## Conclusion

This study contributes to our understanding of how PHNs perceive the challenges of caring for LPIs within the community setting. Given these findings, we believe more research needs to be done to identify the complex health care needs of LPIs and their parents in the community setting. More studies would lead to the development of research-informed practice guidelines; and encourage a more integrated approach of health care delivery from hospital to home, in order to improve the quality of care for families of LPIs.

## Abbreviations

LPI: Late preterm infants
PHN: Public health nurse
